# CCAAT/Enhancer-Binding Protein Homologous Protein (CHOP) Deficiency Attenuates Heatstroke-Induced Intestinal Injury

**DOI:** 10.1007/s10753-021-01577-x

**Published:** 2021-11-29

**Authors:** Yan Cao, Maiying Fan, Yanfang Pei, Lei Su, Weiwei Xiao, Fang Chen, Jie Huang, Xiehong Liu, Zhengtao Gu, Zhongwei Zhang, Fangfang Yuan, Yu Jiang, Xiaotong Han

**Affiliations:** 1 Department of Emergency, Hunan Provincial People’s Hospital, The First Affiliated Hospital of Hunan Normal University, Hunan Province, No.61 Western Jiefang Road, Changsha, 410005 China; 2 Department of Intensive Care Medicine, General Hospital of Southern Theatre Command of PLA, Guangzhou, China; 3 Institute of Emergency Medicine, Hunan Provincial Key Laboratory of Emergency and Critical Care Metabonomics, Hunan Provincial People’s Hospital, The First Affiliated Hospital of Hunan Normal University, Hunan Province, No.61 Western Jiefang Road, Changsha, 410005 China; 4 Department of Treatment Center For Traumatic Injuries, The Third Affiliated Hospital of Southern Medical University, Guangzhou, China; 5 Department of Hematology, The Third Xiangya Hospital of Central South University, Changsha, China

**Keywords:** endoplasmic reticulum stress, heatstroke, CHOP, intestinal injury, 4-phenylbutyrate.

## Abstract

The intestine is one of the main target organs involved in the pathological process of heatstroke. CCAAT/enhancer-binding protein homologous protein (CHOP) is involved in endoplasmic reticulum (ER) stress-induced apoptosis. This study aimed to explore the role of CHOP in heatstroke-induced intestinal injury and potential therapy. An *in vitro* heat stress (HS) model using Caco-2 cells was employed. We observed the role of CHOP in apoptosis-mediated intestinal epithelial cell injury secondary to HS by evaluating cell viability, lactate dehydrogenase release, apoptosis levels, and GRP78, PERK, ATF4, CHOP, Bcl-2, and BAX mRNA and protein expression. To further study the role of CHOP in HS-induced intestinal barrier dysfunction, we assessed transepithelial electrical resistance, paracellular tracer flux, ultrastructure of tight junctions, and protein expression of ZO-1 and occludin. Male wild-type mice and CHOP knockout mice were used for *in vivo* experiments. We evaluated serum d-lactate and diamine oxidase levels, histopathological changes, intestinal ultrastructure, and ZO-1 and occludin protein expression. HS activated the PERK-CHOP pathway and promoted apoptosis by upregulating BAX and downregulating Bcl-2; these effects were prevented by CHOP silencing. Intestinal epithelial barrier function was disrupted by HS *in vitro* and *in vivo*. CHOP silencing prevented intestinal barrier dysfunction in Caco-2 cells, whereas CHOP knockout mice exhibited decreased intestinal mucosal injury. The ER stress inhibitor 4-phenylbutyrate (4-PBA) prevented HS-induced intestinal injury *in vitro* and *in vivo*. This study indicated that CHOP deficiency attenuates heatstroke-induced intestinal injury and may contribute to the identification of a novel therapy against heatstroke associated with the ER stress pathway.

## INTRODUCTION

With global warming, the morbidity and mortality of heatstroke have increased in recent years [1, 2]. Clinically, heatstroke is characterized by extreme hyperthermia (core temperature ≥ 40 °C), central nervous system dysfunction, and multiple organ failure. The intestine is one of the primary target organs involved in the pathophysiological process of heatstroke. The intestine is considered to be the largest reservoir of bacteria and toxins in the body. Approximately 300–500 different types of bacteria are present in the human intestine [3]. Endotoxins and pathogens can enter the circulatory system of the body through an impaired intestinal barrier, inducing endotoxemia and multiple organ dysfunction syndrome (MODS) [4]. However, the underlying mechanisms of intestinal injury in heatstroke remain poorly understood.

The mechanical barrier of the intestine is composed of intestinal epithelial cells, tight junctions (TJs), and a mucus layer covering the epithelial cell surface [5]. The intestinal mucosal epithelium provides a vital barrier against luminal pathogens. Endoplasmic reticulum (ER) is an important intracellular organelle that plays a pivotal role in maintaining cellular homeostasis. Recently, it has been reported that an increase in markers of ER stress was observed in the intestinal epithelia of patients with active inflammatory bowel disease (IBD), indicating that ER stress is relevant to the pathogenesis of IBD [6]. However, little is known about the role of ER stress in mediating the intestinal epithelial barrier dysfunction caused by heatstroke.

Apoptosis is programmed cell death. The integrity of the intestinal structure depends on the balance of intestinal epithelial cell proliferation and apoptosis [7]. Prior studies have found that excessive apoptosis can damage the intestinal epithelial barrier, and the apoptosis index is positively correlated with permeability [8]. In recent years, ER stress has become a hot topic in the field of apoptosis. CCAAT/enhancer-binding protein homologous protein (CHOP), one of the most sensitive factors involved in the regulation of ER stress, is a pivotal marker of ER stress-mediated apoptosis [9]. The upstream regulators of CHOP are three ER transmembrane proteins involving protein kinase R-like endoplasmic reticulum kinase (PERK), activating transcription factor 6 (ATF6) and inositol-required enzyme-1α (IRE1α)[9]. Following the dissociation of glucose-regulated protein 78 (GRP78) from these three transmembrane proteins, three types of unfolded protein responses (UPRs) are triggered by ER stress. Among these pathways, the PERK/eukaryotic translation initiation factor 2α/activating transcription factor4 (PERK/eIF2ɑ/ATF4) signaling pathway is more important for CHOP protein expression compared with the other two pathways [10, 11]. Thus, we hypothesized that CHOP may represent a key factor in regulating heatstroke-induced intestinal barrier dysfunction.

4-Phenylbutyrate (4-PBA), an inhibitor of ER stress, is currently approved by the Food and Drug Administration (FDA) for the treatment of patients with urea cycle disorders (UCDs). 4-PBA functions as a chemical chaperone that prevents misfolded protein aggregation and relieves ER stress [12]. Recent studies have explored the potential therapeutic effects of 4-PBA in various experiments. For instance, in lipopolysaccharide (LPS)-induced acute lung injury (ALI) models, 4-PBA plays a protective role by inhibiting ER stress and autophagy [13]. In a mouse model of alcoholic hepatitis, 4-PBA prevented CHOP upregulation and inflammasome activation in the proximal small intestine [14]. The potential effect of 4-PBA on heatstroke and the underlying mechanisms require further study.

Therefore, our experiment was designed to investigate the role of CHOP in the pathogenesis of intestinal barrier dysfunction in the context of heatstroke and the therapeutic effect of the ER stress inhibitor 4-PBA. We applied two well-established models by performing heat stress (HS) on Caco-2 cells and mice to mimic heatstroke *in vitro* and *in vivo*.

## MATERIALS AND METHODS

### Cell Culture and Groups

In our study, tunicamycin (TM) was used as an ER stress inducer [15]. Human gut–derived Caco-2 cells (Procell Life Science & Technology Co., Ltd., Wuhan, China) were used as a model. These cells have been approved as suitable for studying intestinal epithelial barrier function. The cells were cultured in Dulbecco’s modified Eagle’s medium (DMEM) supplemented with 10% fetal bovine serum (FBS) at 37 °C in a humidified atmosphere of 5% CO_2_. At approximately 80% confluence, the cells were divided into the following eight groups: control group, HS group, CHOP-siRNA + HS group, control-siRNA + HS group, CHOP-plasmid + HS group, control-plasmid + HS group, 4-PBA + HS group, and TM + HS group.

### Cell Treatment

Caco-2 cells were divided into the 8 groups as mentioned above. In the control group, the cells were grown in an incubator at 37 °C. In the HS group, the cells were grown in an incubator at 43 °C for 2 h. In addition, the culture medium was refreshed, and the cells were further incubated at 37 °C for an additional 6 h. A previous study showed that the peak of cell injury and apoptosis was achieved 6 h after HS [16]. In the CHOP-siRNA + HS group, the cells were transfected with CHOP-siRNA 48 h before HS. In the control-siRNA + HS group, the cells were transfected with a control-siRNA 48 h before HS. In the CHOP-plasmid + HS group, the cells were transfected with a CHOP overexpression plasmid 48 h before HS. In the control-plasmid + HS group, the cells were transfected with a control plasmid 48 h before HS. In the 4-PBA + HS group, the cells were pretreated with 5 mmol/L 4-PBA 1 h before exposure to HS. Finally, in the TM + HS group, the cells were pretreated with 5 µg/ml TM 1 h before exposure to HS.

### Cell Transfection

Caco-2 cells were transfected with CHOP-siRNA (GenePharma, Shanghai, China) or the CHOP overexpression plasmid (Tsingke, Beijing, China) according to the manufacturer’s instructions. The cells were seeded in 6-well plates 24 h before transfection in antibiotic-free medium. The cell confluence rate before transfection was approximately 70%. Then, 5 μl of CHOP-siRNA, 5 μl of control siRNA, 5 μl of the CHOP overexpression plasmid, or 5 μl of control plasmid was diluted in 250 μl of Opti-MEM (Gibco, Carlsbad, CA, USA). Five microliters of Lipofectamine 2000 (Invitrogen, Carlsbad, CA, USA) was also diluted in 250 μl of Opti-MEM for each reaction. The siRNA or plasmid solution was mixed gently with Lipofectamine 2000 and incubated for 20 min at room temperature. Then, the transfection complexes were added to 6-well plate cells at 500 µl per well. The culture medium was refreshed after culture for 6 h at 37 °C in a 5% CO_2_ incubator.

The sense sequences (5′ to 3′) were as follows: CHOP sense, GCU GAG UCA UUG CCU UUC UTT and CHOP antisense, AGA AAG GCA AUG ACU CAG CTT; negative control sense, UUC UCC GAA CGU GUC ACG UTT; and negative control antisense, ACG UGA CAC GUU CGG AGA ATT.

### Cell Viability Assays

The cell survival rate of each group was determined using a 3-(4,5-dimethylthiazol-2-yl)-2,5-diphenyltetrazolium bromide (MTT) kit (Beyotime Co, Shanghai, China) according to the manufacturer’s instructions.

### Lactate Dehydrogenase Analysis

Lactate dehydrogenase (LDH) is released when the cell membrane is damaged. Thus, LDH release was used to assess cell damage. LDH enzymatic activity was detected using an LDH kit following the manufacturer’s instructions (JianChen Co, Nanjing, China).

### Hoechst 33258 Staining

Hoechst 33258 staining was performed to observe the morphological changes of cellular nuclei. The cells of each group were washed with PBS, fixed with 4% polyformaldehyde for 10 min, and then stained with Hoechst 33258 (Beyotime, Shanghai, China) solution for 10 min at room temperature in the dark. Morphological changes in the nuclei were then examined under a fluorescence microscope (Eclipse Ti-SR, Nikon Corporation, Tokyo, Japan). Under fluorescence microscopy, the living cellular nuclei stained by Hoechst dye present diffuse and uniform fluorescence, whereas the nuclei of dead cells exhibit blue dense staining or fragmentary dense staining.

### Flow Cytometry Analysis

Caco-2 cell apoptosis in each group was measured by flow cytometry based on Annexin V FITC-propidine iodide (PI) double staining (Invitrogen, Carlsbad, CA, USA). According to the manufacturer’s instructions, the cells were digested with trypsin without ethylenediaminetetraacetic acid (EDTA) and centrifuged to harvest. The harvested cell number was approximately 1 × 10^5^ cells. Next, Annexin V-FITC/PI staining was performed according to the protocol provided with the kit (Invitrogen, Carlsbad, CA, USA). After staining, the proportion of apoptotic cells was analyzed by flow cytometry (BD, New Jersey, USA). The apoptotic rate was calculated as the percentage of early apoptotic cells in the lower right quadrant.

### qRT–PCR Analysis

Total RNA was extracted from the Caco-2 cells of each group using Trizol (Invitrogen, Carlsbad, CA, USA). RNA was transcribed into cDNA using a PrimeScriptTM RT Reagent Kit (Takara, Shiga, Japan). The PCR mixture (20-µl final volume per reaction) was prepared. Amplification was performed by quantitative real-time PCR with the TB Green Premix Ex TaqTM II kit (Takara, Shiga, Japan). The primer sequences are reported in Table 1. Actin served as the endogenous reference gene to normalize the data.

**Table 1:** Primer Sequences (5′-3′)

Gene		Primer
GRP78	Forward	GAATTCCTCCTGCTCCTCGT
	Reverse	CAGCATCATTAACCATCCTTTCG
PERK	Forward	ACGATGAGACAGAGTTGCGAC
	Reverse	ATCCAAGGCAGCAATTCTCCC
eIF2ɑ	Forward	AAGCATGCAGTCTCAGACCC
	Reverse	GTGGGGTCAAGCGCCTATTA
ATF4	Forward	ACAAGACAGCAGCCACTA
	Reverse	CTTACGGACCTCTTCTATCAG
CHOP	Forward	GGAAACAGAGTGGTCATTCCC
	Reverse	CTGCTTGAGCCGTTCATTCTC
Bcl-2	Forward	GGTGGGGTCATGTGTGTGG
	Reverse	CGGTTCAGGTACTCAGTCATCC
Bax	Forward	TCACTGAAGCGACTGATGTCCC
	Reverse	ACTCCCGCCACAAAGATGGTC
Actin	Forward	ACCCTGAAGTACCCCATCGAG
	Reverse	AGCACAGCCTGGATAGCAAC

### Transepithelial Electrical Resistance Measurement

Caco-2 cells were plated on collagen-coated membrane Transwell inserts (3-µm pore size filters, Corning, USA). The transepithelial electrical resistance (TEER) of cells was measured with an electrical resistance system (EVOM, World Precision Instruments, Berlin, Germany). The TEER was calculated by normalizing to the initial values and was expressed as percentages of the initial resistance values.

### Paracellular Tracer Flux Assay

In this assay, FITC-dextran (2.5 mg/mL, Sigma–Aldrich, USA) was added to the upper chamber 2 h before the experimental endpoint. After incubation, the fluorescence concentration of FITC-dextran in the medium of the lower chamber was measured by fluorescence at Ex 490 nm/Em 520 nm (TecanGENios reader, Tecan Group Ltd., CH).

### Animals

CHOP^−/−^ mice on a C57BL/6 background were purchased from Model Organism (Shanghai, China). Considering that estrogen can increase survival during heatstroke by relieving inflammatory responses and cardiovascular dysfunction [17], only male mice were used in the study. Male wild-type (WT) and CHOP^−/−^ mice (12 weeks old) were used. The animal experiments were approved by the Ethics Committee of Hunan Provincial People’s Hospital, The First Affiliated Hospital of Hunan Normal University, Changsha, China. All animals were housed in a controlled environment at a constant temperature of 21 ± 2 °C with a 12-h light/dark cycle.

### Heatstroke Procedures

The mice were randomly divided into six groups: the WT + sham group (*n* = 6), CHOP^−/−^  + sham group (*n* = 6), WT + PBA + sham group (*n* = 6), WT + HS group (*n* = 6), CHOP^−/−^ + HS group (*n* = 6), and WT + PBA + HS group (*n* = 6). We applied HS to mice to mimic heatstroke *in vivo*. To generate the heatstroke model, mice were transferred to an artificial climate chamber with an environmental temperature of 35.5 ± 0.5 °C and humidity of 60 ± 5%. Rectal temperature (Tc) was continuously measured using a mercury thermometer every 15 min. The time point at which the Tc reached 42 °C was taken as the point of heatstroke onset [18, 19]. After the Tc reached 42 °C, the animals were allowed to recover at room temperature (24 ± 0.5 °C). In the WT + PBA + HS group, the WT mice were pretreated with 4-PBA (100 mg/kg, ip) before HS. In the WT + sham or CHOP^−/−^  + sham group, the mice were maintained under sham-heated conditions at a temperature of 24 ± 0.5 °C and humidity of 35 ± 5%. The mice in the WT + PBA + sham group were pretreated with 4-PBA (100 mg/kg, ip) before being maintained under the same conditions. A previous study demonstrated that the average survival time of mice with heatstroke was approximately 6 h even when cooling treatment was applied [20]. Therefore, this time point was used for our subsequent experiment. The mice were sacrificed at this time point under anesthesia, and serum and the ileum were isolated.

### Serum d-Lactate and Diamine Oxidase Levels

Serum samples were collected and measured using a corresponding enzyme-linked immunosorbent assay (ELISA) kit (Nanjing Jiancheng Co. Ltd., Nanjing, China) to detect serum d-lactate (d-LA) and diamine oxidase (DAO) following the manufacturer’s instructions.

### Histopathology

Ileum specimens from each group were fixed in 10% neutral-buffered formalin. The specimens were then embedded in paraffin blocks, sectioned at 5–7 µm, and stained with hematoxylin and eosin (H&E). The histopathological changes in ileal tissue were assessed under light microscopy.

### Ultrastructural Observation by Transmission Electron Microscopy

The ultrastructural changes in Caco-2 cells or ileal tissues were observed by transmission electron microscopy. Caco-2 cell or ileum tissue specimens were fixed with 2.5% glutaraldehyde and then postfixed with 1% osmium tetroxide. Then, the specimens were dehydrated using a graded ethanol series (concentrations of 50%, 70%, 80%, 90%, and 100%) into pure acetone. After dehydration, the specimens were embedded with graded mixtures of acetone and SPI-PON812 resin and then polymerized for 12 h at 45 °C and 48 h at 60 °C. Finally, ultrathin Sects. (70 nm) were stained and viewed under transmission electron microscopy (Hitachi, Tokyo, Japan).

### Western Blotting

Proteins were extracted from Caco-2 cells or ileal tissues using RIPA buffer (Beyotime, Shanghai, China) to obtain total protein. The protein concentration was detected using a BCA protein assay kit (Applygen Technologies, Inc., Beijing, China). Equal amounts of protein (30 μg) were separated on SDS–PAGE gels and transferred to nitrocellulose membranes (Millipore, Bedford, MA). The membranes were blocked in a 5% skim milk-TBS solution at room temperature for 1 h. Then, the membranes of cell proteins were incubated with diluted primary antibodies against the following proteins: GRP78, PERK, eIF2α, p-eIF2α, and CHOP (Cell Signaling Technology, Danvers, Massachusetts, USA); ATF4, B-cell lymphoma-2 (Bcl-2), and Bcl-2 Associated X Protein (Bax) (Proteintech Group, Inc. Rosemont, Illinois, USA); and zonula occluden-1 (ZO-1) and occludin (Abcam, Massachusetts, United States). The membranes of tissue proteins were incubated with diluted primary antibodies against ZO-1 and occludin. Afterward, the membranes were washed thrice in PBS/Tween-20 for 5 min with shaking. The membranes were then incubated with the secondary antibody (Zhongshan Inc., China) for 2 h at room temperature. The membranes were developed using a GE ImageQuant LAS 500 (GE Healthcare, USA). Quantification of the digitized images of the Western blot bands was performed using ImageJ software (National Institutes of Health, Bethesda, Maryland, USA).

### Statistical Analysis

All the data were presented as the mean ± standard deviation, and differences were analyzed with one-way analysis of variance (ANOVA) among groups using SPSS statistical software (SPSS for Windows, version 19.0, Chicago, IL). A value of *P* < 0.05 was considered statistically significant.

## RESULTS

### The Effects of CHOP Silencing, CHOP Overexpression, an ER Stress Inhibitor, or an ER Stress Inducer on Caco-2 Cell Morphology, Viability and Damage Under HS

**Fig. 1:**
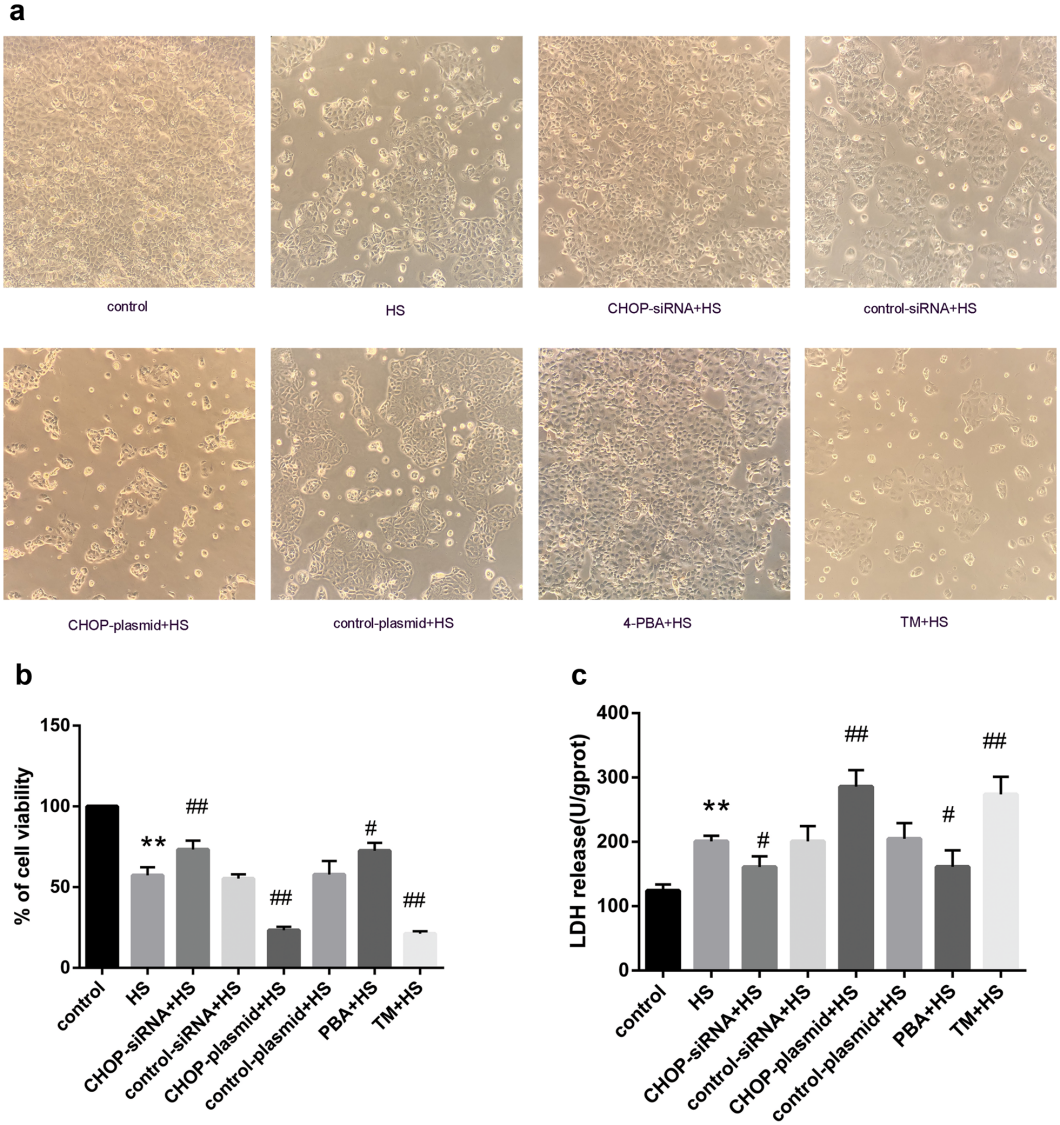
Changes in cellular morphology, viability, and damage levels under HS in different experimental groups. **a** Morphological changes in Caco-2 cells as observed under a microscope (magnification × 100). **b** MTT assay was used to detect cell viability. **c** LDH release analysis was used to detect the level of cell damage. Significant differences are indicated as follows: **P < 0.01 and *P < 0.05 versus the control group; ##*P* < 0.01 and #*P* < 0.05 versus the HS group.

An inverted microscope was used to observe the morphological changes of Caco-2 cells in each group. As shown in Fig. 1a, normal Caco-2 cells adhered to the wall and grew in a monolayer. HS induced cellular shrinkage, an increase in floating dead cells, and a decrease in living cells. After transfection with CHOP-siRNA or pretreatment with 4-PBA, the cell morphology was close to normal. Following transfection with the CHOP overexpression plasmid or pretreatment with TM, cellular shrinkage was aggravated, and numerous floating dead cells were observed. To investigate the survival rate of Caco-2 cells after HS, we measured cell viability using the MTT assay. As shown in Fig. 1b, HS significantly decreased cell viability. After transfection with CHOP-siRNA or pretreatment with 4-PBA before HS, the cell viability increased compared with that of the HS group. Following transfection with the CHOP overexpression plasmid or pretreatment with TM, cell survival significantly decreased compared with that of the HS group. Additionally, as shown in Fig. 1c, the LDH level was increased after HS. Transfection with CHOP-siRNA or pretreatment with 4-PBA decreased LDH levels after HS. However, the LDH level in the CHOP overexpression group or TM group was even greater than that in the HS group.

### CHOP Activation Plays an Important Role in HS-Induced Apoptosis, and ER Stress Inhibition Can Alleviate HS-Induced Apoptosis

**Fig. 2:**
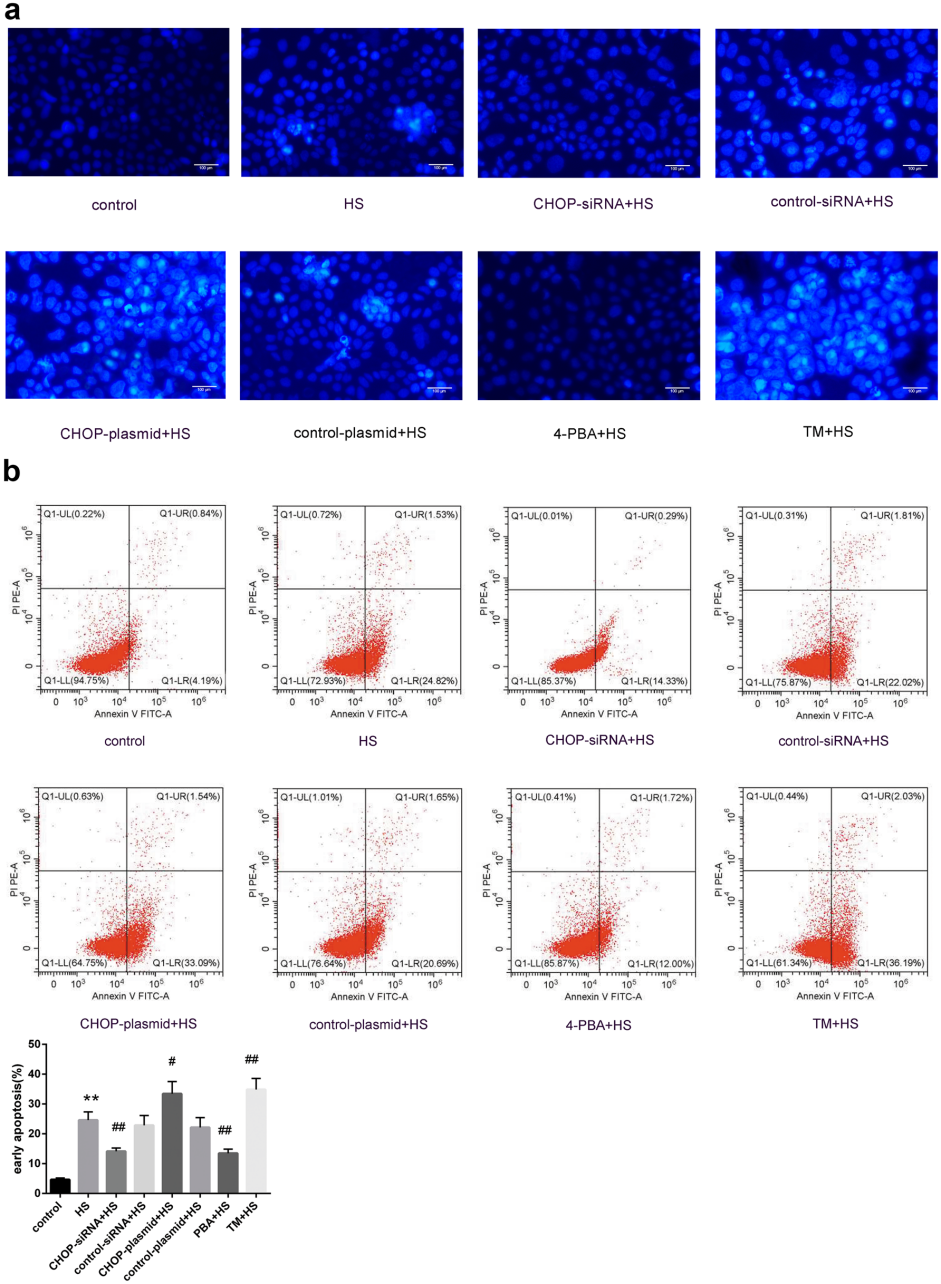
Hoechst 33258 fluorescence nuclear staining and Annexin V-FITC/PI double-staining flow cytometry were used to detect the changes in apoptosis levels in different experimental groups. **a** Hoechst 33258 fluorescence nuclear staining was used to detect nuclear apoptotic morphological changes (magnification × 200). **b** Annexin V-FITC/PI double-staining flow cytometry was used to detect the levels of apoptosis. Significant differences are indicated as follows: **P < 0.01 and *P < 0.05 versus the control group; ##*P* < 0.01 and #*P* < 0.05 versus the HS group.

Morphological changes in apoptotic nuclei were observed by Hoechst 33258 staining, whereas apoptosis was detected by flow cytometry with Annexin V-FITC/PI double staining. As shown in Fig. 2a, the nuclei of living cells stained by Hoechst exhibited diffuse and uniform fluorescence, while the nuclei of dead cells revealed condensed or fragmented staining. Compared with the control group, a large number of apoptotic nuclei with bright blue staining were present in the HS group. CHOP-siRNA or 4-PBA significantly prevented HS-induced apoptosis. In contrast, CHOP overexpression or TM further induced apoptosis.

As shown in Fig. 2b, the apoptosis level was detected using flow cytometry with Annexin V-FITC/PI double staining. We observed that HS significantly increased Caco-2 cell apoptosis levels. In the CHOP-siRNA and 4-PBA groups, the number of apoptotic cells significantly decreased compared with the HS group. CHOP overexpression and TM pretreatment exacerbated the apoptosis induced by HS. These results indicate that CHOP activation plays a crucial role in HS-induced apoptosis and that the ER stress inhibitor 4-PBA has a remarkable protective effect on Caco-2 cells under HS.

### The Effects of CHOP Silencing, CHOP Overexpression, an ER Stress Inhibitor, or an ER Stress Inducer on mRNA and Protein Expression Levels of Components of the HS-Activated PERK-CHOP Pathway

**Fig. 3:**
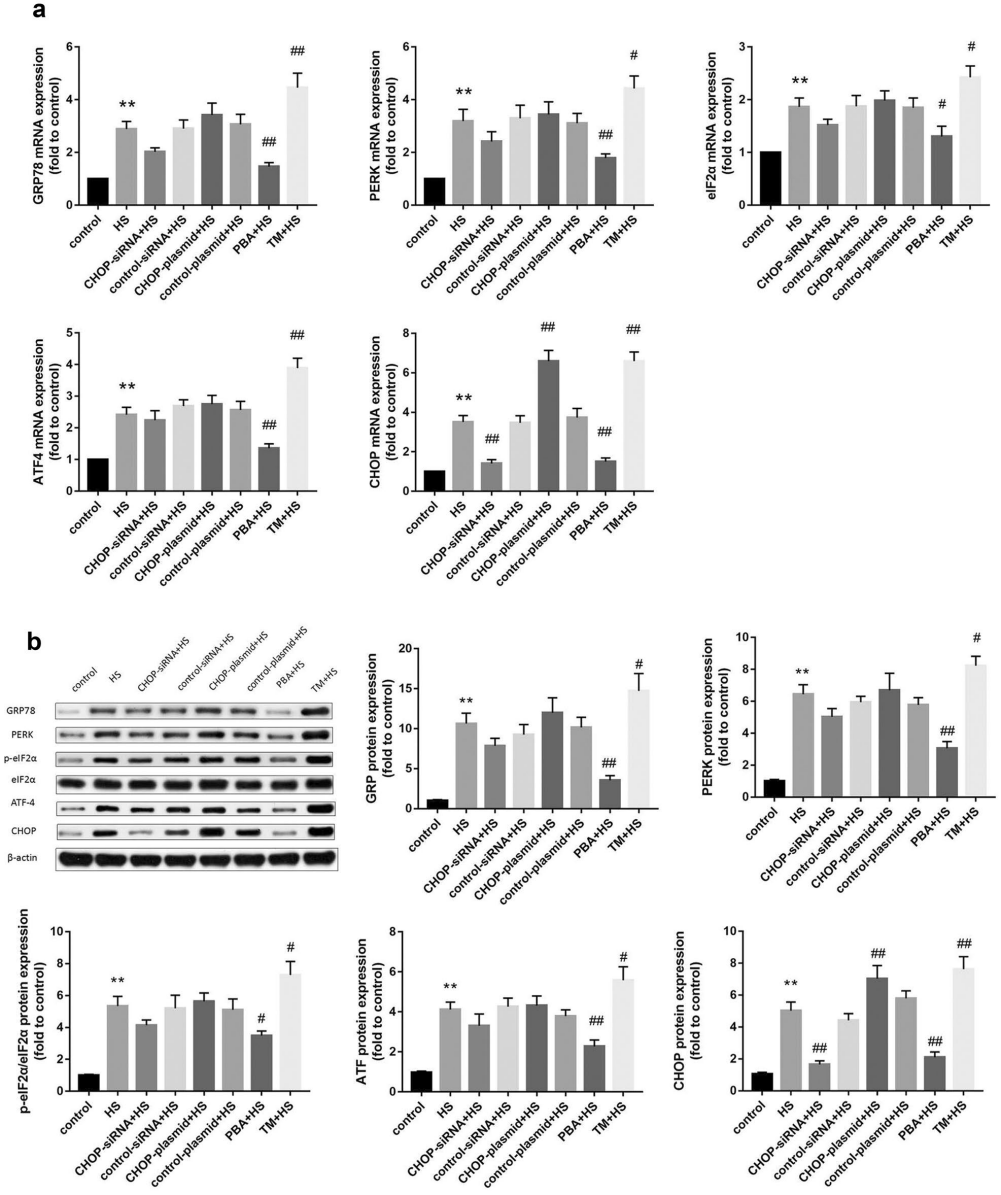
**a** qRT–PCR was used to detect the mRNA expression of PERK-CHOP signaling pathway–related factors in different experimental groups. **b** Western blotting was used to detect GRP78, PERK, p-eIF2α, ATF4, and CHOP protein expression. The graphs show the relative band densities of the target protein to β-actin normalized against the control group. Significant differences are indicated as follows: **P < 0.01 and *P < 0.05 versus the control group; ##*P* < 0.01 and #*P* < 0.05 versus the HS group.

In our present study, we used qRT*–*PCR and Western blotting to confirm the role of CHOP in the PERK-CHOP pathway. As shown in Fig. 3a, HS significantly increased GRP78, PERK, eIF2ɑ, ATF4, and CHOP mRNA expression levels. As shown in Fig. 3b, GRP78, PERK, ATF4, and CHOP protein expression levels in the HS group were significantly increased compared with those in the control group. eIF2ɑ phosphorylation was enhanced after HS, whereas total eIF2ɑ protein levels showed no significant changes.

Additionally, CHOP-siRNA significantly reduced CHOP expression at both the mRNA and protein levels. Conversely, following CHOP overexpression plasmid transfection, a significant increase in CHOP mRNA and protein expression was detected. Interestingly, CHOP silencing reduced GRP78, PERK, eIF2ɑ, and ATF4 mRNA expression compared with that in the HS group, albeit without statistical significance. Subsequently, a reduction in GRP78, PERK, p-eIF2ɑ, and ATF4 protein levels was also detected without statistical significance.

In addition, pretreatment with 4-PBA before HS significantly decreased the mRNA and protein expression of PERK-CHOP pathway members compared with that in the HS group, whereas pretreatment with TM before HS had the opposite effect, as shown in Fig. 3a, b.

### The Effects of CHOP Silencing, CHOP Overexpression, an ER Stress Inhibitor, or an ER Stress Inducer on Bcl-2 and Bax mRNA and Protein Expression

**Fig. 4:**
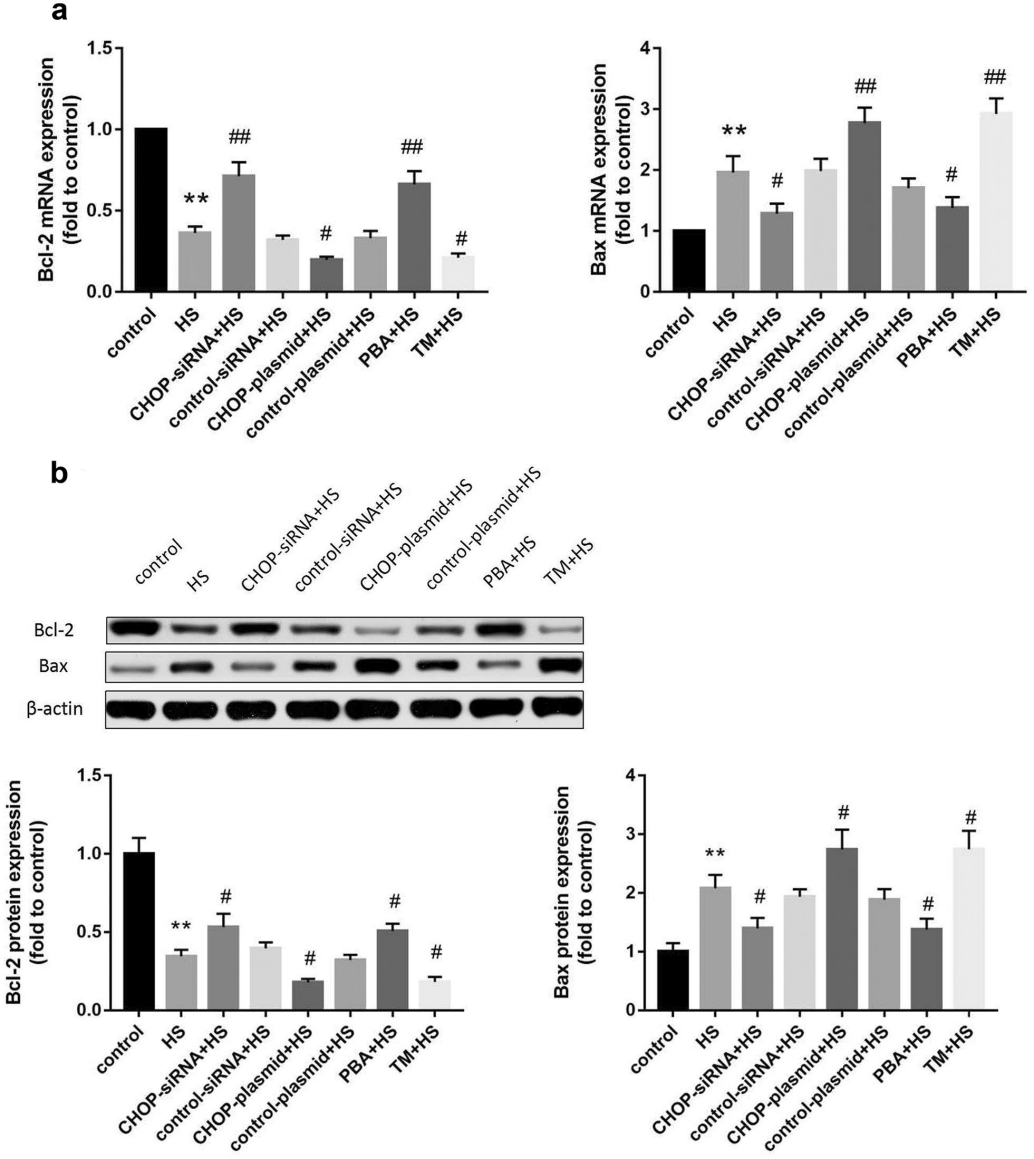
**a** qRT–PCR was used to detect Bcl-2 and Bax mRNA expression in different experimental groups. **b** Western blotting was used to detect Bcl-2 and Bax protein expression. The graphs show the relative band densities of the target protein to β-actin normalized against the control group. Significant differences are indicated as follows: **P < 0.01 and *P < 0.05 versus the control group; ##*P* < 0.01 and #*P* < 0.05 versus the HS group.

Bcl-2 is an antiapoptotic protein, while Bax is a proapoptotic protein. To explore the role of CHOP in modulating Bcl-2 family members in response to HS, we used qRT–PCR and Western blotting to measure the mRNA and protein expression levels of Bcl-2 and Bax. As shown in Fig. 4a, b, Bax mRNA and protein expression levels in the HS group were significantly upregulated after HS, whereas Bcl-2 mRNA and protein expression levels were markedly decreased. However, a significant reduction in Bax and an increase in Bcl-2 at both the mRNA and protein expression levels were observed when CHOP was silenced before HS. Following CHOP overexpression plasmid transfection before HS, a marked increase in Bax and a reduction in Bcl-2 mRNA and protein expression levels were detected. These findings suggested that CHOP is a key factor that mediates ER stress–induced apoptosis by modulating Bcl-2 family members in response to HS.

Furthermore, as shown in Fig. 4a, b, 4-PBA significantly increased Bcl-2 and decreased Bax mRNA and protein expression levels compared with the HS group. Pretreatment with TM before HS significantly decreased Bcl-2 and increased Bax the mRNA and protein expression levels compared with the HS group. These results demonstrated that the inhibition of ER stress has a protective effect on HS-induced apoptosis by upregulating Bcl-2 and downregulating Bax.

### CHOP Deficiency or ER Stress Inhibition Prevents Intestinal Barrier Dysfunction Induced by HS Both* In Vitro* and *In Vivo*

Increasing evidence has suggested that apoptosis is related to the increased expression of CHOP during ER stress [21]. In our current study, we identified that CHOP overexpression aggravates HS-induced apoptosis, whereas CHOP silencing or 4-PBA pretreatment protects against HS-induced apoptosis. Next, we investigated the role of CHOP silencing or 4-PBA pretreatment on intestinal barrier function *in vitro* and *in vivo*.

**Fig. 5:**
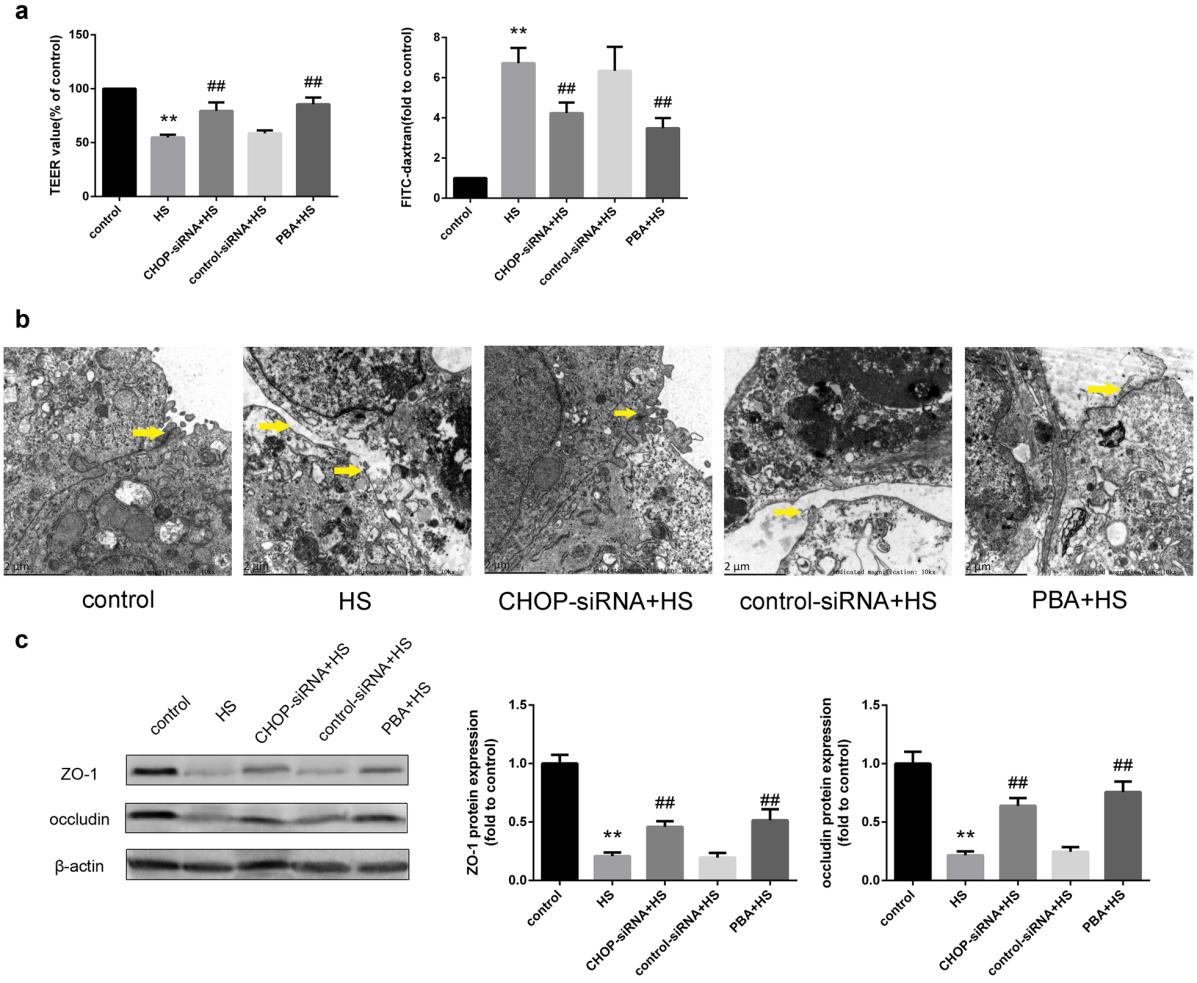
Silencing CHOP by siRNA prevented intestinal barrier dysfunction induced by HS in Caco-2 cells. **a** TEER value is presented relative to the control group (%TEER). The FITC-dextran value is presented relative to the control group. **b** Morphological ultrastructure of TJs under TEM. Yellow arrows indicate TJs (magnification × 10,000). **c** Western blotting was used to detect ZO-1 and occludin protein expression. The graphs show the relative band densities of the target protein to β-actin normalized against the control group. Significant differences are indicated as follows: **P < 0.01 and *P < 0.05 versus the control group; ##*P* < 0.01 and #*P* < 0.05 versus the HS group.

Epithelial barrier integrity and permeability of Caco-2 cells were measured by TEER and FITC-dextran flux. As shown in Fig. 5a, HS resulted in a significant reduction in TEER compared with the control group. CHOP silencing or 4-PBA pretreatment prevented the reduction in TEER induced by HS. Consistent with the TEER results, a significant increase in the paracellular permeability of FITC-dextran is noted in Fig. 5a, and CHOP silencing or 4-PBA pretreatment reversed the increase in paracellular permeability. As demonstrated by transmission electron microscopy in Fig. 5b, heat exposure destroyed the tight junction ultrastructure in Caco-2 monolayers. In the control group, intact tight junctions were noted between intestinal epithelial cells. In the HS group, cell and organelle structures were dramatically destroyed, the intercellular spaces were widened, and tight junctions were obviously opened. However, CHOP silencing or 4-PBA pretreatment greatly improved the ultrastructure of cells, and tight junctions were more continuous. As shown in Fig. 5c, Western blotting revealed that ZO-1 and occludin protein expression decreased after HS, whereas CHOP silencing or 4-PBA pretreatment obviously increased ZO-1 and occludin expression.

**Fig. 6:**
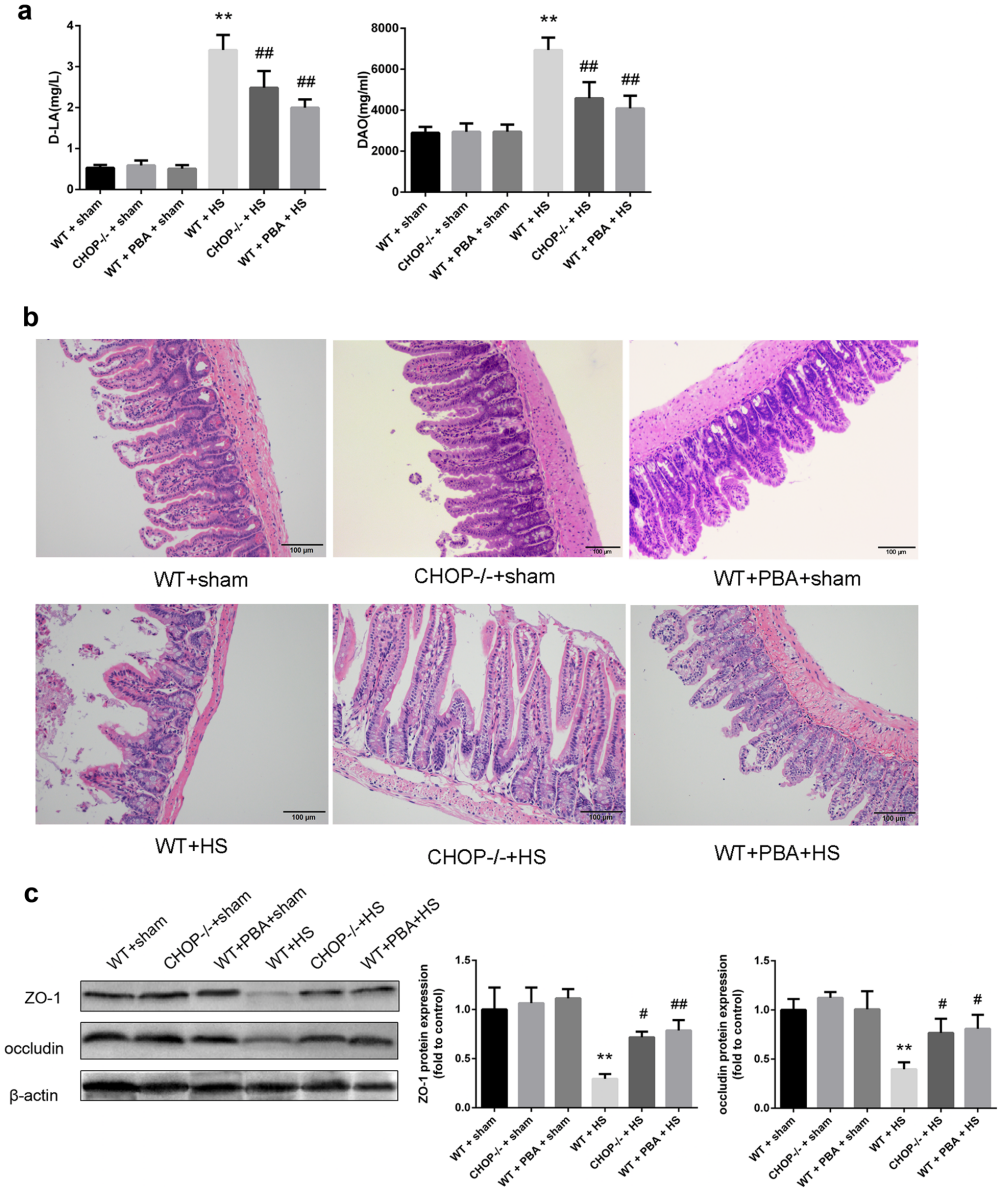
CHOP knockout prevented intestinal barrier dysfunction induced by HS in mice. **a** Serum d-LA and DAO concentrations. **b** Histopathological changes in the ileum were observed by H&E staining (magnification × 200). **c** Western blotting was used to detect ZO-1 and occludin protein expression. The graphs show the relative band densities of the target protein to β-actin normalized against the WT + sham group. Significant differences are indicated as follows: **P < 0.01 and *P < 0.05 versus the WT + sham group; ##*P* < 0.01 and #*P* < 0.05 versus the WT + HS group.

**Fig. 7:**
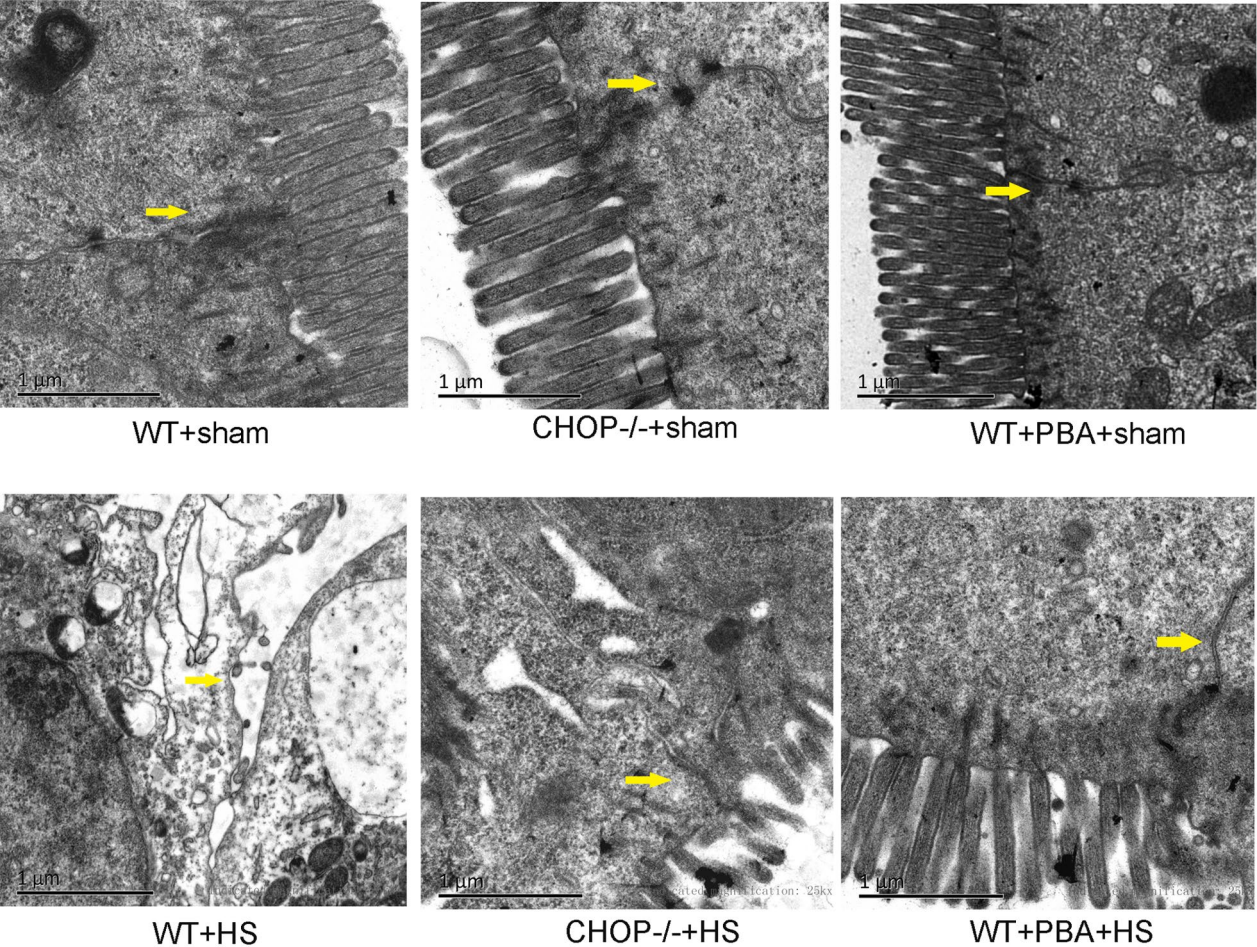
Morphological ultrastructure of TJs in mice. Yellow arrows indicate TJs (magnification × 25,000).

In an *in vivo* study, increases in serum D-LA and DAO levels were observed in WT mice with heatstroke, and these increases were significantly inhibited in CHOP^−/−^ mice or mice pretreated with 4-PBA, as shown in Fig. 6a. In addition, H&E staining showed that mice exposed to HS presented profound damage to the epithelium of the small intestine, which manifested as extensive destruction of the villi and inflammatory cell infiltration, as shown in Fig. 6b. Heatstroke-induced pathological changes were also significantly reduced in CHOP^−/−^ mice pretreated with 4-PBA. As obtained from transmission electron microscopy in Fig. 7, we observed extreme destruction of tight junctions in the ileum of mice with heatstroke compared with the control tissue, which was significantly alleviated in CHOP^−/−^ mice or upon pretreatment with 4-PBA. Next, we examined the expression levels of the tight junction proteins ZO-1 and occludin in the ileum. As shown in Fig. 6c, heatstroke decreased ZO-1 and occludin protein expression in WT mice, which was reversed in CHOP^−/−^ mice or upon pretreatment with 4-PBA.

## DISCUSSION

Intestinal barrier dysfunction plays a critical role in heatstroke progression, but the underlying mechanisms remain poorly understood. To our knowledge, the current study identifies a novel mechanism underlying heatstroke-induced intestinal injury. Our results indicate that CHOP mediates intestinal epithelial apoptosis and barrier dysfunction induced by heatstroke. 4-PBA prevents apoptosis and improves the integrity of the intestinal barrier, underscoring its utility as a potential treatment for heatstroke.

As one of the key transcription factors in ER stress, CHOP is widely expressed in a variety of mammalian body cells. Under normal physiological conditions, CHOP expression is extremely low. However, CHOP expression is significantly increased in response to ER stress, thereby activating a series of downstream apoptotic molecules to induce apoptosis and participate in the occurrence and development of various pathological processes [21]. Apoptosis is a type of programmed cell death that is implicated in intestinal epithelial cell death [22]. The dynamic balance of intestinal epithelial cell proliferation and apoptosis maintains the homeostasis of the intestinal barrier. Under pathological conditions, repair and proliferation of the intestinal mucosa are difficult to achieve, and excessive apoptosis of intestinal mucosal cells will inevitably cause damage to the intestinal barrier [23]. In the pathophysiology of MODS secondary to heatstroke, the gastrointestinal tract is one of the first onset organs. Severe heatstroke can lead to increased intestinal epithelial apoptosis and impaired intestinal mucosal barrier followed by an endotoxemia-induced inflammation cascade and systemic inflammatory response syndrome (SIRS), which ultimately leads to MODS or even death [24]. In our study, we first established a cellular model of heatstroke using Caco-2 cells and observed the role of CHOP in apoptosis-mediated intestinal epithelial injury. We observed that CHOP was activated in Caco-2 cells after HS. Reductions in CHOP expression via transfection with siRNA increased cell viability and decreased apoptosis, whereas CHOP overexpression significantly decreased cell survival and increased apoptosis. These results revealed that CHOP was essential in HS-induced Caco-2 cell apoptosis.

CHOP is regulated by PERK, ATF6, and IRE1, but the PERK/eIF2ɑ/ATF4 signaling pathway plays a more important role in CHOP activation compared with the other two UPR pathways [25]. In this pathway, PERK stimulates eIF2α phosphorylation to enhance the translation of ATF4, thereby increasing CHOP transcription. Bcl-2 and Bax are downstream target genes of the PERK-CHOP apoptosis signaling pathway [26]. Our findings showed that HS increased the mRNA and protein expression of factors involved in the PERK-CHOP pathway in Caco-2 cells. By silencing CHOP before HS, Bax mRNA and protein expression levels were reduced, whereas Bcl-2 mRNA and protein expression levels were increased. However, CHOP overexpression upregulated Bax expression and downregulated Bcl-2 expression. Our data strongly support our hypothesis that CHOP plays a pivotal role in HS-induced apoptosis by regulating Bcl-2 and Bax.

Interestingly, after CHOP silencing, the expression of the ER stress chaperone proteins GRP78, PERK, eIF2ɑ, and ATF4 was decreased, albeit without statistical significance. Similarly, in a study on the mechanism of rifampicin-associated liver damage, GRP78, PERK, and ATF4 expression was also downregulated after CHOP silencing [27]. Regarding the effect of silencing CHOP on GRP78 and the upstream factors of CHOP, such as PERK, eIF2ɑ, and ATF4, previous studies have reported the presence of a feedback loop in the ER stress pathway [28]. Thus, we hypothesize that these upstream and downstream kinases may affect each other through a feedback loop in the same signaling pathway.

In addition, the results also indicated that pretreating Caco-2 cells with the ER stress inhibitor 4-PBA can significantly attenuate HS-induced changes in cell morphology and apoptosis by inhibiting PERK-CHOP pathways, subsequently downregulating the proapoptotic protein Bax and upregulating the antiapoptotic protein Bcl-2. In contrast, the ER stress-inducer TM aggravated Caco-2 cell apoptosis. Our results explore the potential therapeutic effects of 4-PBA in heatstroke.

Next, we explored the role of CHOP in regulating intestinal barrier function *in vitro* and *in vivo*. Maintenance of intestinal barrier integrity relies on a variety of mucosal structural components, such as tight junctions, which form zonula occludentes at the apical surface between cells [29]. It has been reported that extreme heat induces intestinal permeability and TJ protein disruption [30]. A previous study demonstrated that CHOP^−/−^ mice are protected from bile duct ligation (BDL)-induced disruption of intestinal barrier function [31]. Our results showed that HS induced morphological injuries and ultrastructural damage (especially to TJs) of the intestinal mucosa, accompanied by an increase in intestinal permeability and disruption of epithelial integrity *in vitro* and *in vivo*. CHOP silencing significantly attenuated the HS-induced decrease in TEER and increase in FITC-dextran release in Caco-2 cells by ameliorating the changes in tight junction structure and regulating TJ protein expression. *In vivo* studies with CHOP^−/−^ mice further demonstrated that CHOP deficiency obviously alleviated HS-induced intestinal tissue damage and barrier dysfunction. In addition, our data showed that 4-PBA repaired dysfunction of the intestinal barrier following HS both *in vitro* and *in vivo*, indicating that ER plays a central role in the maintenance of intestinal mucosal homeostasis.

In conclusion, we have demonstrated for the first time that CHOP deficiency attenuates heatstroke-induced intestinal mucosal damage and barrier dysfunction. Here, we have provided strong evidence that targeting ER stress represents a novel therapeutic strategy for heatstroke.

## Data Availability

All data generated or analyzed during the current study are included in this article.

## Code Availability

No custom algorithm, software, or code was used in the present work.
